# Palaeobiology of red and white blood cell-like structures, collagen and cholesterol in an ichthyosaur bone

**DOI:** 10.1038/s41598-017-13873-4

**Published:** 2017-10-23

**Authors:** Chloé Plet, Kliti Grice, Anais Pagès, Michael Verrall, Marco J. L. Coolen, Wolfgang Ruebsam, William D. A. Rickard, Lorenz Schwark

**Affiliations:** 1 WA-Organic and Isotope Geochemistry, Department of Chemistry, The Institute for Geoscience Research, Curtin University, Curtin, WA 6845 Australia; 2 CSIRO CESRE, Mineral resources, Kensington, WA 6151 Australia; 3 Department of Organic Geochemistry, Institute of Geoscience, Christian Albrechts University, Kiel, 24118 Germany; 4 Advanced Resource Characterisation Facility, John de Laeter Centre, Curtin University, Curtin, WA 6845 Australia

## Abstract

Carbonate concretions are known to contain well-preserved fossils and soft tissues. Recently, biomolecules (*e*.*g*. cholesterol) and molecular fossils (biomarkers) were also discovered in a 380 million-year-old concretion, revealing their importance in exceptional preservation of biosignatures. Here, we used a range of microanalytical techniques, biomarkers and compound specific isotope analyses to report the presence of red and white blood cell-like structures as well as platelet-like structures, collagen and cholesterol in an ichthyosaur bone encapsulated in a carbonate concretion from the Early Jurassic (~182.7 Ma). The red blood cell-like structures are four to five times smaller than those identified in modern organisms. Transmission electron microscopy (TEM) analysis revealed that the red blood cell-like structures are organic in composition. We propose that the small size of the blood cell-like structures results from an evolutionary adaptation to the prolonged low oxygen atmospheric levels prevailing during the 70 Ma when ichthyosaurs thrived. The δ^13^C of the ichthyosaur bone cholesterol indicates that it largely derives from a higher level in the food chain and is consistent with a fish and cephalopod diet. The combined findings above demonstrate that carbonate concretions create isolated environments that promote exceptional preservation of fragile tissues and biomolecules.

## Introduction

Dinosaur fossils, even with the most beautifully preserved anatomy, generally lack soft tissues such as fibrous or cellular remains as well as biomolecules or molecular fossils. However, over the last three decades, several studies have shown that fragile tissues and molecules can be preserved over surprisingly long periods of time (tens of millions of years)^1–8^.

Heme-derived porphyrins were detected in a blood engorged mosquito from the Middle Eocene^1^. More recently, red blood cell (RBC)-like structures, along with amino acids associated with collagen-like fibres, were also found in 75 million-year-old dinosaur bones^8^. The latter finding was remarkable considering the fact that the bone fragments were not particularly well preserved, which is in agreement with models suggesting that preservation of biomolecules and soft tissues in the fossil record is more common than previously thought^8–11^. Collagen fibres were also reported *in-situ* in a 195 million-year-old dinosaur^7^.

**Figure 1:**
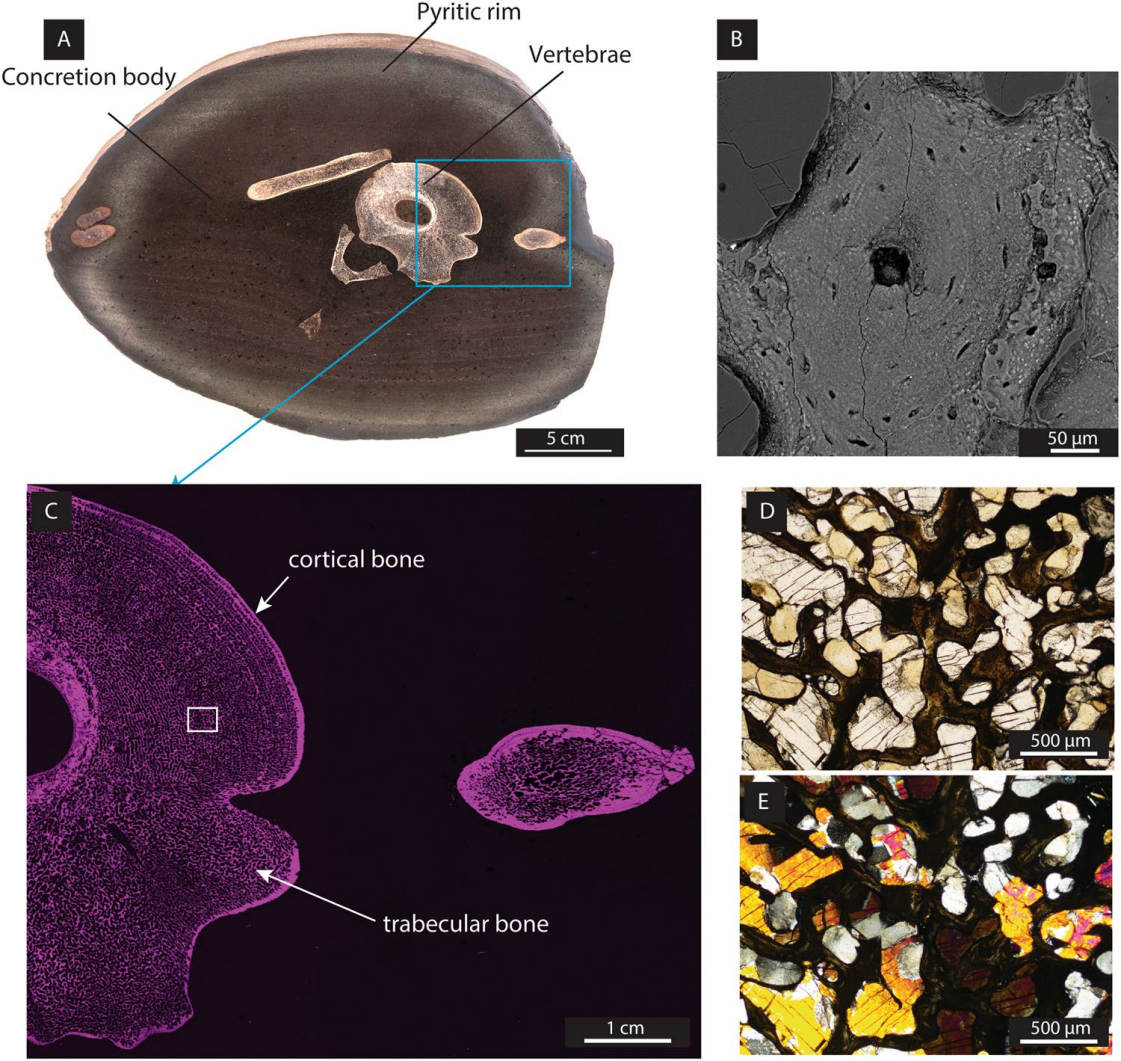
Morphology, mineralogy and chemical composition of ichthyosaur bones within a carbonate concretion. (**A**) Photographic image of a polished section of the bone-containing concretion. The vertebra served as the main nucleus which triggered the microbial degradation processes leading to the concretion. The rim contains a high amount of pyrite (observed by XRD, optical microscopy and SEM) in contrast to the concretion body. (**B**) Backscattered electron image of a Haversian system, including Haversian canal, osteocytes and lamellae from the bone. (**C**) Microbeam XRF elemental mapping of phosphorus (magenta) showing that phosphorus is relatively more abundant in the bones than in the concretion. The blue  square represents the area where the Haversian system was imaged. (**D** and **E**) Optical imaging on a thin section using ppl (**D**) and xpl (**E**) showing the minerals filling the porosity of the bone: equant sparry calcite (CaCO_3_) and barite (BaSO_4_). BaSO_4_ was identified by its mineral properties (clear colour and 90° cleavages) and high birefringence as well as by elemental distribution using microbeam XRF (Figure S1).

Here, we investigated an ichthyosaur vertebra (*Stenopterygius*) of Lower Toarcian age (~182.7 Ma), which has been preserved through encapsulation in a carbonate concretion (Fig. 1). The sample was collected from the renowned Posidonia Shale Konservat Fossil Lagerstätte in SW-Germany. Ichthyosaurs thrived in the Mesozoic era; they evolved following the largest mass extinction to have affected life on our planet (during the Olenekian Stage of the Early Triassic, between 251.1 Ma and 247.2 Ma)^12,13^ and became extinct at the end-Cenomanian (93.9 Ma)^13^.

Generally, during the Jurassic, ichthyosaur falls in shallow waters had low preservation potential for tissues and biomolecules due to the presence of a specialised consortium of degraders^14^. However, in the Lower Toarcian, when the *Harpoceras falciferum* zone was deposited, the preservational environment in epicontinental seas was excellent for tissues and biomolecules due to water column stratification and strong euxinic conditions in the bottom waters^15,16^. Under these euxinic conditions, organic matter-rich mudstones were deposited and the diagenetic formation of carbonate concretions was common^17^. Such carbonate concretions often contain fossils^18,19^ or, in some exceptional cases, even biomolecules^20,21^.

The aim of this study was to investigate the potential of carbonate concretions to preserve microscopic soft tissue and biomolecules from a vertebra of the ichthyosaur *Stenopterygius*. A combined approach using *in-situ* imaging techniques and molecular investigations was applied to study this carbonate concretion and encapsulated fossil. Here, we report the oldest RBC, white blood cell (WBC) and platelet-like structures, >100 Myr older than in a previous report^8^ as well as the second oldest occurrences of collagen fibres^7^ and cholesterol^20^.

## Results and Discussion

### Encapsulation of an ichthyosaur vertebra in a concretion

A range of imaging techniques was applied to a polished section of the vertebra (Fig. 1A–E). A selection of three-dimensional samples from the vertebra cortical and trabecular bones was taken. Both cortical and trabecular bones display a homogenous structure. Early mineralisation of concretions around the decaying organic matter may occur within weeks or months^22^. During this early encapsulation, the formation of a tight carbonate cement prevented the bone from further microbial degradation and inhibited exchange of fluids with the surrounding environment. The concretion body is composed of microspar calcite and small (~10 µm) dispersed euhedral crystals of pyrite. The outer rim of the concretion is rich in pyrite. No septaria were observed within the concretion, which further supports the limited post-depositional exchange with the diagenetic environment. Therefore, early post mortem encapsulation led to preservation of the bone tissue in the concretion.

### Bone structure and elemental mapping

Microbeam XRF mapping of phosphorus (P) showed that P is relatively more abundant in the bone fragments than within the concretion (Fig. 1C), and helped to distinguish the cortical (*i*.*e*. compact) bone from the trabecular (*i*.*e*. spongy) bone. The high primary porosity (*e*.*g*. up to 65%) of vertebra bones has been reported previously in ichthyosaurs^23^. We calculated a porosity of the same range (estimated at ~60%) in the trabecular bone (Fig. 1C), where pores have been predominantly cemented by calcite (Fig. 1D and E). Elemental mapping (Ba, S) (Figure S1) and optical imaging (Fig. 1D and E) revealed a bone compartment cemented with trace element-enriched barite (BaSO_4_), a feature often observed in bones deposited under anoxic conditions where trace elements may be mobilised from a black shale^24^.

Examination of the internal bone structure of the ichthyosaur, using backscattered electron imaging, revealed remarkable preservation of fossilised 250 µm-diameter secondary osteons (Haversian system), known to be involved in mature bone remodelling and renewal. Within the osteons, a number of osteocytes and lamellae are visible (Fig. 1B). Osteocytes play a predominant role in the synthesis of collagen and regulate osteoblast function as well as biomineralisation of bones (*e*.*g*.^25^).

### Red and white blood cells, platelets and collagen fibres in an ichthyosaur

**Figure 2:**
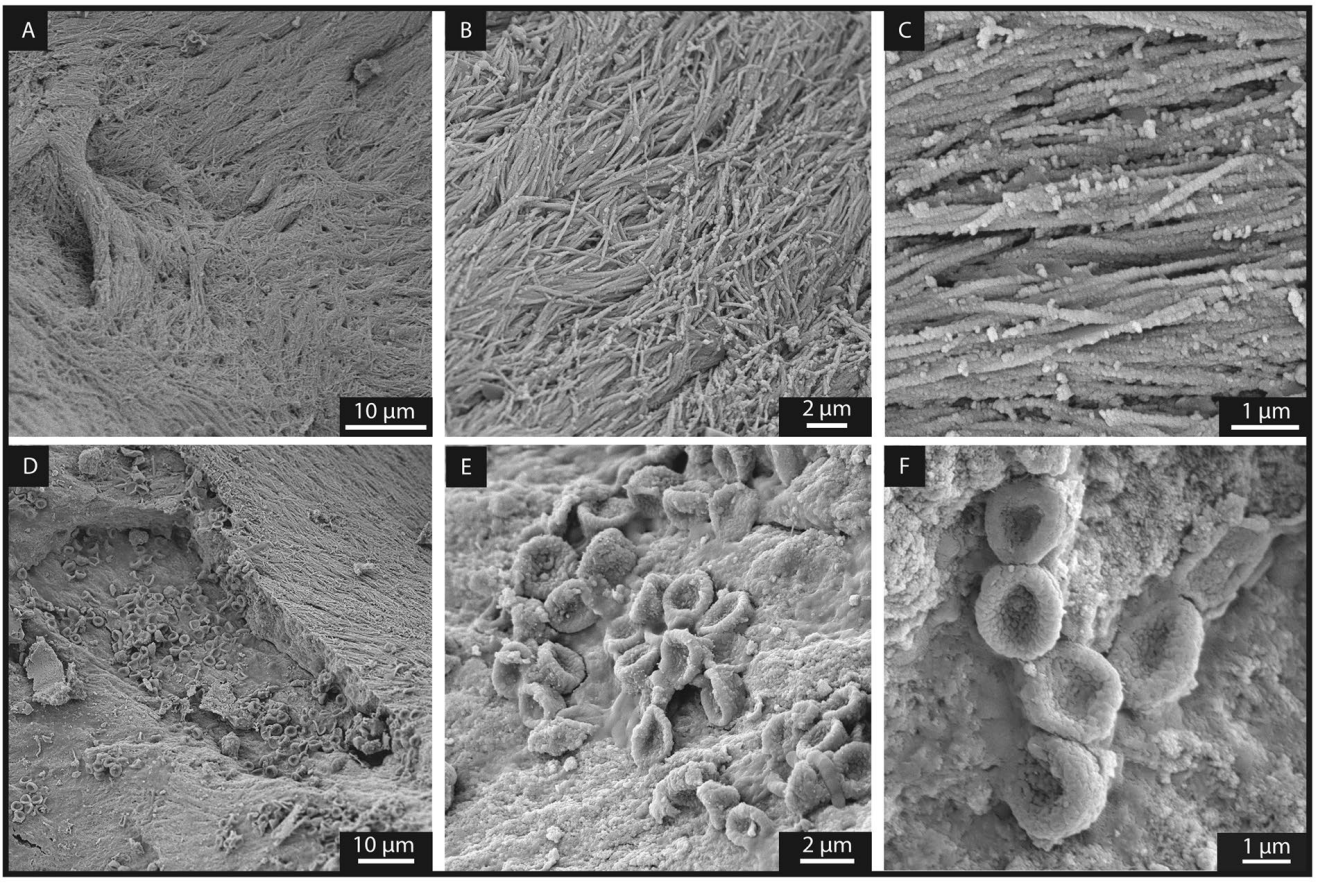
Secondary electron images of the trabecular bone following the removal of sparry calcite by light acetic acid treatment revealing exceptionally well-preserved soft tissues. (**A** to **C**) Represent collagen fibres^8^ with increasing magnification. (**D** to **F**) Reveal RBC-like structures with increasing magnification.

**Figure 3:**
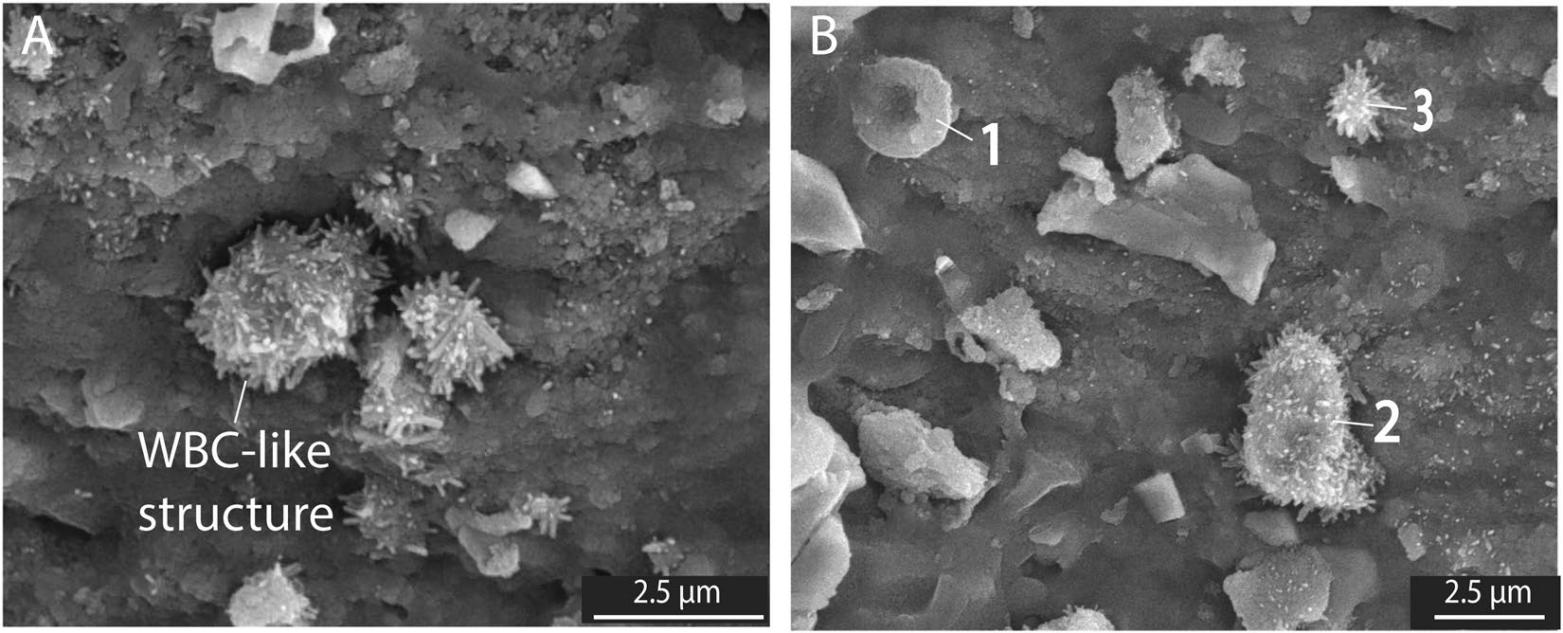
Secondary electron  images of the trabecular bone following the removal of sparry calcite by light acetic acid revealing soft tissues. (**A**) Presence of WBC-like structures. (**B**) 1) indicates a RBC-like structure, 2) indicates a WBC-like structure and 3) indicates a platelet-like structure.

Scanning electron microscopy (SEM) analyses were performed on samples from the trabecular and cortical bones. Images were acquired after removal of the carbonate filling the bone porosity, as described in Material and Methods. SEM imaging of fossilised soft tissue in the trabecular bone (Fig. 2) revealed intertwined elongated fibres (average width of 160 ± 1 nm; *n* = 88). These fibres show curved geometries and bundles (Fig. 2A–C) which, in size and orientation, resemble modern crocodile collagen (Figure S3). These fibres also are within the diameter range (size comprised between 130 to 250 nm for 30 measurements) of collagen fibres reported in Late Cretaceous dinosaurs^4,8^. In close proximity to these collagen fibres, clusters of concave disks with an average size of 1.95 ± 0.21 µm (*n* = 75), closely resembling RBC-like structures reported from dinosaurs^8^, were observed (Fig. 2D–F). In addition to RBC-like structures, WBC- and platelet-like structures were identified (Fig. 3) based on morphological comparison with modern analogues^26^. However, all these blood cell-like structures are generally four to five times smaller than those identified in modern mammals^27^.

**Figure 4:**
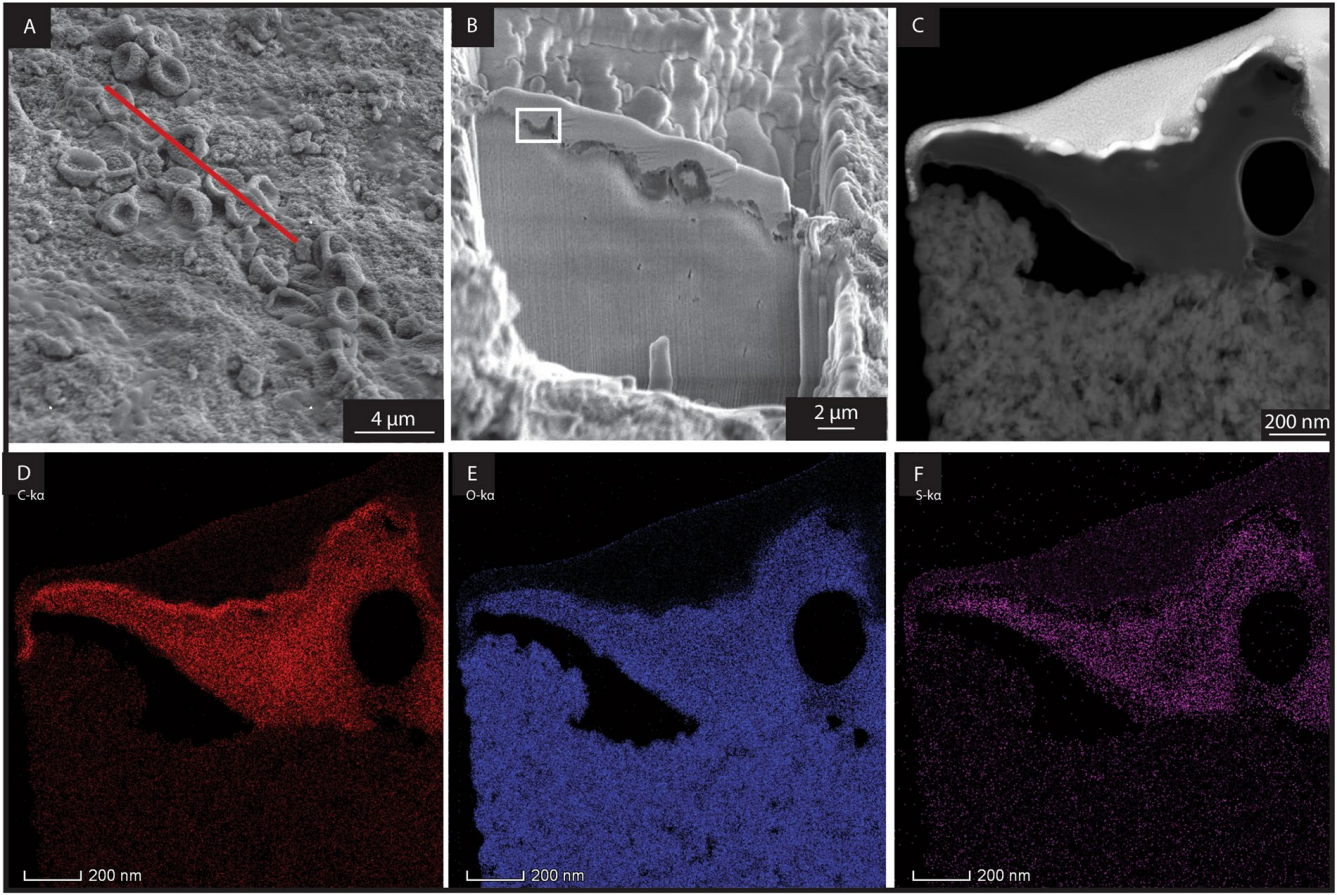
Area extracted by FIB-SEM for TEM analysis. (**A**) Secondary electron image of the trabecular bone showing the presence of RBC-like structures. The TEM foil was extracted from a cross-section showed by the red line. (**B**) Secondary electron image taken during TEM foil preparation showing the cross-section of the foil just prior to lift-out. The white rectangle indicates the area selected for TEM elemental mapping in (**D**,**E** and **F**). (**C**) TEM-HAADF image of a RCB-like structure. (**D**) Carbon (C) distribution of the RCB-like structure by TEM. (**E**) Oxygen (O) distribution of the RCB-like structure. (**F**) Sulfur (S) distribution in the RBC-like structure.

**Figure 5:**
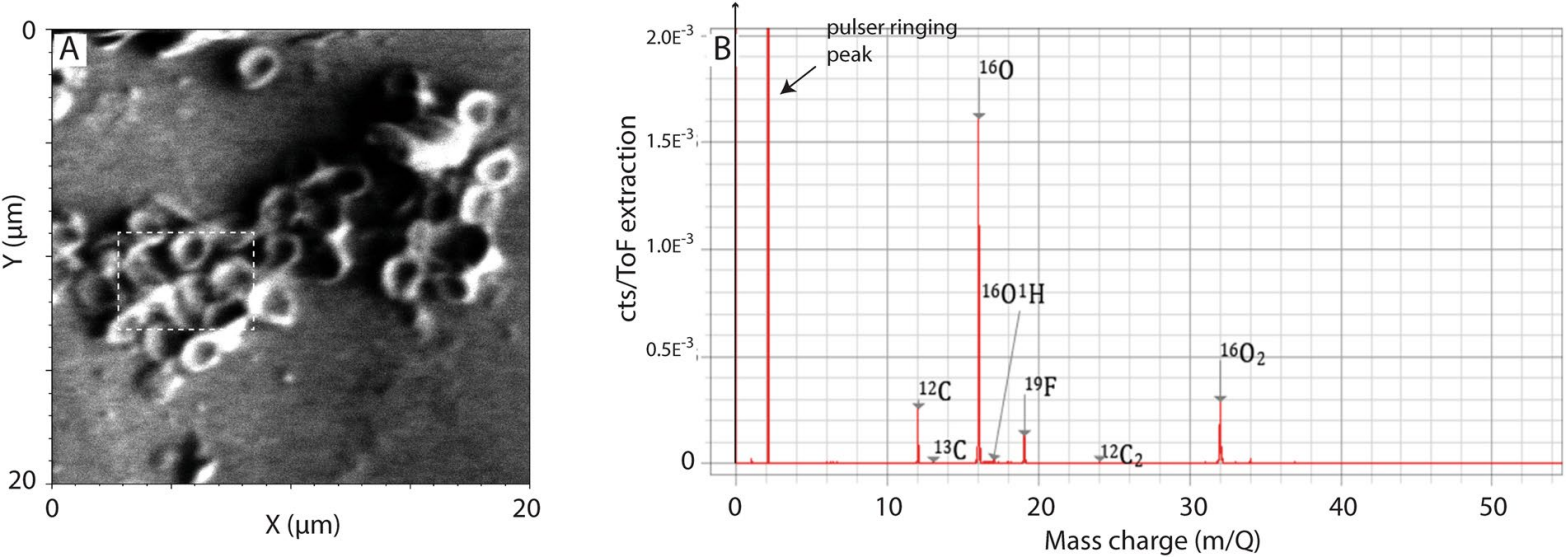
ToF -SIMS analysis of RBC-like structures in the ichthyosaur vertebra. (**A**) Secondary image of the RBC-like structures by ToF-SIMS. The white rectangle correspond to the area where the mass spectra was acquired. (**B**) Negative ions mass spectra showing the presence of C, O and Fluorine (F) specifically associated with the RBC-like structures.

RBC-like structures were isolated and analysed by transmission electron microscopy (TEM), (Fig. 4) which highlighted the presence of both carbon and oxygen in these structures. Time of Flight Secondary Ion Mass Spectrometry (ToF-SIMS) analyses of the RBC-like structures revealed the abundant light isotopes of carbon (^12^C) and oxygen (^16^O), further supporting an organic origin (Figs 5 and S2). Additional evidence for an organic origin is confirmed by the identification of the polar compound Me,Et maleimide (3-ethyl, 4-methyl-pyrrole-2,5-dione) extracted from the bone. Indeed, Me,Et maleimide is a known oxidative degradation product of heme and chlorophyll pigments^28^. It is thus suggested that this maleimide likely derived from heme.

#### Origin of the RBC-like structures

Due to their small size, the RBC-like structures could potentially be interpreted as derived from bacteria. Here, we present several arguments supporting a blood cell origin rather than a bacterial origin. All RBC-, WBC-, and platelet-like structures were exclusively detected in the vertebra bone. This is inconsistent with a bacterial origin, as bacteria would be expected to be present in the vertebra as well as the surrounding concretion (body and rim). In addition, all blood cell-like structures were only revealed on the bones surfaces after removing the carbonate filling the bone porosity. This suggests they were entombed under the carbonate cement since it formed about 183 Ma ago, further supporting that these blood cell-like structures cannot be the result of recent bacterial colonisation. Furthermore, the RBC-like structures are not simply deposited on the bone, but are locally fused into it (Fig. 2D–F), which is consistent with the fact that erythropoiesis (blood cell formation) occurs in medullar bones (*e*.*g*. vertebrae).

Lastly, coccoid shaped bacteria are generally smaller (0.5–2 μm) than the RBC-like structures observed here and they lack a concave shape. The other major bacterial shapes (rods and vibrios) have absolutely no resemblance with the shape of the RBC-like structures. For these reasons, we conclude that the concave-shaped structures show similarities with modern day RBCs. Similarly, the absence of hopanols within the bone suggests that these structures are not of bacterial origin. In addition, the dramatic variation in shape and size of RBCs within a single class of modern animal (e.g. mammals) has been reported since 1875 (as cited by^29,30^). Since the extinction of the dinosaurs (~65 Ma), a rapid evolution and diversification of mammalian species took place, colonising many vacant ecological niches . This rapid evolution and diversification was also reflected in the great variety of size and shape of RBCs in mammals^29,30^. Similarly, during the Mesozoic era which lasted ~187 Myr, reptiles reached their highest diversity and numerous species appeared and became extinct. It seems highly possible that Jurassic reptiles could have also presented diversity in their RBC shape as well as size, in order to efficiently adapt to the surrounding paleoenvironmental conditions. We therefore propose that the small size of these blood cell-like structures observed therein is related to evolutionary adaptation to environmental conditions.

#### Evolutionary adaptation to environmental conditions

Ichthyosaurs evolved during an episode typified by low atmospheric oxygen levels, lasting over 70 million years from the Early Triassic to the Lower Jurassic^31^. We suggest that under the prolonged low oxygen levels in the atmosphere^32–34^, small RBCs could have been favoured because the surface to volume ratio^35^ provides a more efficient oxygen transport and diffusion. For example, mammals living at high altitude have been shown to have excellent adaptation to low oxygen levels based on abundant RBCs of small size^35^. The “bowl-like” shape of the cells resembling RBCs (*i*.*e*. stomatocytes) has been widely reported in disease-related studies of mammalian species with anucleated RBCs^36,37^. However, the study of blood in reptiles is limited, which makes the interpretation of reptilian hematologic data challenging^38,39^.

We hypothesise that the fossil occurrence of small RBC-like structures in ichthyosaurs could be consistent with an oxygen-depleted palaeoenvironment and evolutionary adaptation. This adaptation is supported by the occurrence of RBC-like structures of similar size in terrestrial dinosaurs^8^. Although oxygen concentrations reached today’s levels during the Late Cretaceous^40^, most of dinosaurs’ evolution took place during prolonged periods of low oxygen levels and they lived under the same atmospheric conditions as the ichthyosaurs. In modern fish, RBCs size has been shown to be inversely proportional to aerobic swimming ability^41^. Moreover, a correlation between small RBCs size and high rate of metabolism has also been demonstrated in modern geckos^42,43^. With respect to adaption, we emphasize that *Stenopterygius* is considered to have been one of the fastest marine predators of its time^44^, its cruising speed equivalent to that of modern day dolphin and with a similar morphology^45^. A high degree of RBC aggregation has previously been reported in modern higher athletic species^46^. This metabolic adaptation could potentially explain the clustering of the small RBC-like structures observed in this *Stenopterygius*. In order to sustain the metabolism required for high-speed pursuit predators, the muscular tissue must have been highly efficient and have been supported by a complex blood circulation system, adapted to low-oxygen environment, to provide sufficient oxygen to the lungs of the ichthyosaurs. Given that the bone studied is a medullary bone (*i*.*e*. vertebra), it would yield sufficient bone marrow (see below) to synthesise RBCs. Based on their small size, the fossilised RBC-like structures indicate a fast and efficient oxygen diffusion into the cells, allowing for high pursuit speed and thus providing competitive advantage over slower moving prey.

### Cholesterol in an ichthyosaur

**Figure 6:**
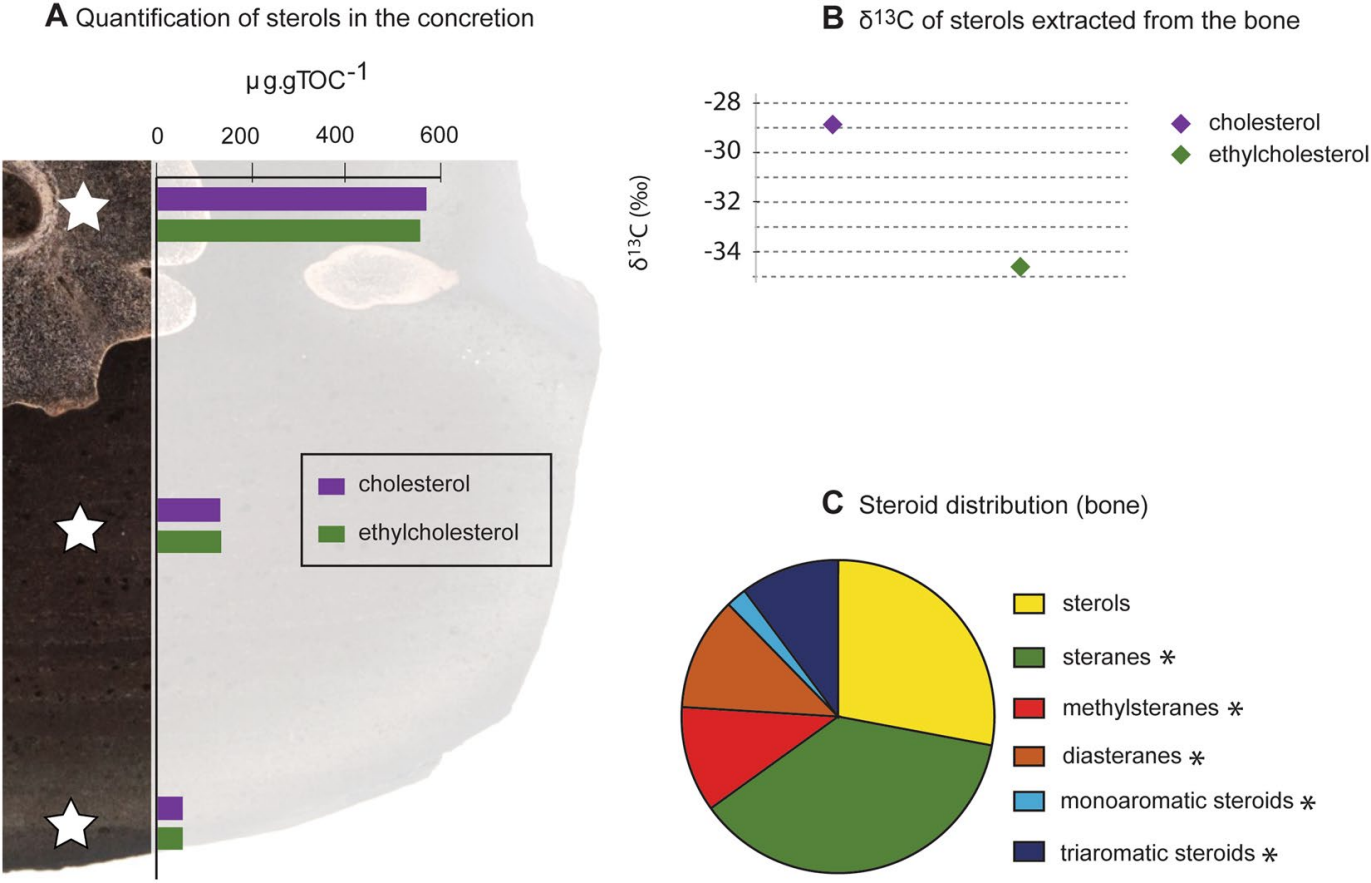
Steroid distribution and compound specific δ^13^C values. (**A**) Sterol distribution within the bone and concretion, showing high concentrations of cholesterol (565 µg/g TOC) and ethylcholesterol (523 µg/g TOC) and lower concentrations in the concretion body and rim. (**B**) δ^13^C values (‰ VPDB) of sterols associated with the bone (cholesterol: −28.9 ± 0.4‰ VPDB; ethylcholesterol: −34.6 ± 0.4‰ VPDB) from the concretion. The error bars are contained within the symbol. (**C**) Relative proportion of compound classes within the fossil, dominated by sterols and steranes representing the end-members of the diagenetic sequence. *Diagenetic end products.

Besides fossilised RBC-, WBC- and platelet-like structures, the ichthyosaur bone contained elevated concentrations of the biomolecule cholesterol (565 µg/g TOC, Fig. 6 and Table S1). It was previously reported that free cholesterol is relatively abundant in the bone marrow^47^ supporting the high amount of neutrally extracted free bone cholesterol in our sample. The bone cholesterol differed in its isotopic carbon composition (−28.9‰ VPDB) compared to ethylcholesterol (−34.6‰ VPDB; Fig. 6). The isotopic discrepancy between these two sterols supports different origin(s). The ^13^C enrichment of the cholesterol by 5.7‰ VPDB indicates that it largely derives from a higher level in the food chain and corroborates a fish and cephalopod diet of the ichthyosaur^48,49^. The ^13^C isotopic composition of the ethylcholesterol is consistent with a source from phytoplankton in the ancient water column. Recently, soft tissue of a crustacean inside a Devonian concretion from the Gogo Formation (Canning Basin, Western Australia) was reported to contain an entire diagenetic continuum of organic molecules with the remarkable co-occurrence of biomolecules and geomolecules, from sterols to triaromatic steroids (including sterenes and diasterenes)^20^. The exceptional preservation of these compounds was attributed to rapid encapsulation by microbially-mediated and eogenetic processes. In our study, steroid end-products of diagenesis were also identified in association with the vertebra (Fig. 6C). However, the absence of sterenes and diasterenes suggests the formation of the concretion within the sediments (corroborated by the preservation of slightly disturbed sedimentary bedding) and was not initiated in the water column^20,21^. The Posidonia Shale Formation and the Gogo Formation concretions were both formed under similar euxinic (H_2_S-rich) conditions and are well known Fossil–Lagerstätten.

## Conclusions

Tight encapsulation of a Jurassic ichthyosaur bone in a diagenetically formed carbonate concretion allowed for the fossilisation of structures showing resemblance to modern day bone collagen, RBCs, WBCs and platelets with a micron-scale preservation. Microanalysis revealed that the RBC-like structures are enriched in organic material.

The rapid encapsulation of the vertebra also led to the preservation of cholesterol, largely derived from the vertebra bone, over a period of ~182.7 million years. These observations highlight the development of a closed environment within carbonate concretions. In such cases, carbonate concretions preserve fossils (structural and cellular) and biomolecules, as well as molecular fossils with an excellent level of detail. This well-preserved primary biomaterial suggests that carbonate concretions could play a major role in the investigation of the palaeobiology of extinct species and in understanding the evolution of life. Based on the small size of the RBCs and their associated high oxygen diffusion capacity, we hypothesise that ichthyosaur must have followed a high-energy life style as a pursuit predator. This would distinguish its hunting strategy from that of an ambush predator life style as assumed for plesiosaurs.

## Material and Methods

### Geological settings and sampling

The investigated sample was recovered from the Toarcian Posidonia Shale Formation at the HOLCIM Cement quarry of Dotternhausen (SW-Germany). The concretion was collected shortly after blasting within the quarry and stored in the dark at room temperature before a transverse slice was cut across the oval shaped concretion (Fig. 1A), just before analyses.

Following the global Toarcian transgression, the black shale host sediment was deposited in the SW-German sub-basin, on the epicontinental Western Tethyan Shelf^50–52^. The sub-basin experienced high algal productivity coupled with restrictions in water circulation leading to stratification of the water column and development of anoxic to euxinic bottom waters, which favoured the deposition of organic matter-rich black shales^16,51,52^.

### Sample preparation

Three 1 cm-slices were cut from the central area of the lens shaped concretion, perpendicular to the horizontal bedding. One slice was polished for microbeam X-Ray Fluorescence (XRF) (Bruker M4 TORNADO^TM^) elemental mapping. A thin section of the vertebra was prepared from the second slice. From the third slice, three samples were taken from i) vertebra, ii) concretion body presenting sedimentary bedding and iii) concretion rim for organic geochemical and compound specific isotope analyses (CSIA). Each sample was cleaned in an ultrasonic bath using a mixture of dichloromethane: methanol (DCM: MeOH) at 9:1 (v/v) (three times × 20 min) to remove any surface contaminants. Fossil bone samples were crushed and mm-sized pieces of bone were collected and treated with 1 M acetic acid solution immediately prior to SEM imaging. The remainder of the samples was pulverised using a Rocklabs benchtop ring mill (BTRM) in a pre-annealed zircon mill. Pre-annealed quartz sand was pulverised and analysed as a procedural blank for organic geochemical techniques. Mineralogy was determined using aliquots of pulverised samples for X-ray diffraction (XRD).

A modern crocodile leg sample was obtained from Mahogany Creek Distributors (Perth, Australia). The bones were cut and isolated from the flesh. A sample was then left to dry in the oven for 24 h (50 °C, below the denaturation threshold of native hydrated collagen fibrils^53^) and treated with concentrated H_2_O_2_ (48 h). The oxidised flesh and bone marrow were removed using forceps and the bone was left to dry at room temperature.

### Mineralogy

XRD analyses on powdered samples were performed using a Bruker-AXS D8 Advance Diffractometer with CuKα radiation and a LynxEye position sensitive detector. The data were collected from 7.5 to 90° 2Ө, with a nominal step size of 0.015° and a collection time of 0.7 seconds per step. Crystalline phases were identified using the Search/Match algorithm, DIFFRAC.EVA 3.1 (Bruker-AXS) to search the Powder Diffraction File.

### Imaging methods

#### Porosity estimation

The estimation of the trabecular porosity of the ichthyosaur vertebra has been determined through digital point counting on a recursive grid to two times 200 points and stabilisation of the point count distribution plot. The overall trabecular porosity evaluated was estimated at 59.5%.

#### Microbeam XRF mapping

A Bruker M4 TORNADO™ Micro-XRF equipped with a rhodium target X-ray tube operating at 50 kV and 500 nA and an XFlash® silicon drift X-ray detector was used for elemental mapping of the polished slice of the Toarcian concretion samples. Maps were created using a 25 µm spot size on a 25 µm raster with dwell time of 5 ms per pixel.

#### Scanning Electron Microscopy (SEM)

Both modern crocodile and fossil ichthyosaur bone samples were coated using a Quorum Q150T ES coating unit. A carbon layer of approximately 25 nm was applied and as samples were charging, an additional thin coating of gold (3–5 nm) was applied.

The bone samples were examined using a Tescan Mira-3 Field Emission Gun Scanning Electron Microscope (FEG-SEM). The instrument was operated with an accelerating voltage 5 kV and a beam current of approximately 200 pA. The images acquired were collected using an Everhart-Thornley Secondary Electron (SE) detector.

#### Focused Ion Beam Scanning Electron Microscope (FIB-SEM)

The sample was examined using a Tescan Lyra FIB-SEM. A small fragment of the bone was mounted onto an aluminium stub and coated with gold. A cross-sectional lamella covering a number of RBC-like structures was extracted using standard FIB-SEM lift out techniques, mounted onto a copper grid and thinned to ~100 nm, followed by a low kV (2 kV) ‘clean up’ routine to remove surface damage.

#### Time of flight secondary ion mass spectrometry (ToF-SIMS)

ToF-SIMS was performed during microstructural analysis using a Tescan Lyra. The instrument is fitted with a TOFWERK ToF-SIMS detector and uses the Ga^+^ ion beam from the FIB as the primary ion source. Analysis was performed over a 20 µm × 20 µm area to a depth of approximately 400 nm using a 20 kV primary ion energy and a current of 500 pA. Negative ions were collected and a mass spectrum was derived from a volume containing only RBC-like structures to reveal their chemical composition.

#### Transmission Electron Microscopy (TEM)

Microstructural analysis and elemental mapping of the FIB-SEM prepared lamella was carried out using high angle annular dark field scanning transmission electron microscopy (HAADF-STEM, FEI Talos F200x TEM/STEM with Super-X EDS Detectors) at 200 kV.

### Lipid biomarker analyses

Toarcian samples were subject to Soxhlet extraction with a mixture of DCM:MeOH (9:1, 72 hrs). Activated copper turnings were added to remove elemental sulfur. An aliquot of each total lipid extract was adsorbed onto activated silica gel (160 °C, >24 hrs). Each aliquot was then separated using column chromatography with a small column containing activated silica gel (5 cm × 0.5 cm i.d.) into five fractions. i) The aliphatic hydrocarbon fraction was eluted using 2 mL of *n-*hexane; ii) the aromatic hydrocarbon fraction was eluted with 2 mL *n-*hexane:DCM (4:1); iii) the ketone and fatty acid methyl esters fraction was eluted with 2 mL DCM; iv) the sterol fraction was eluted with 2 mL DCM:ethyl acetate (4:1) and v) the polar fraction was eluted using DCM:MeOH (7:3).

Sterols were derivatised using bis(trimethylsilyl)-trifluoroacetamide (BSTFA) and anhydrous pyridine (for 100 µg, 60 µL BSTFA, 40 µL pyridine) heated at 70 °C for 30 min and dried under a nitrogen purge. The fractions were dissolved in *n-*hexane and analysed using gas chromatography-mass spectrometry (GC-MS). Semi-quantitative analyses of the sterol fractions were carried out using external calibration with a derivatised cholesterol standard. Procedural blanks were performed throughout.

GC-MS analyses were performed using a Hewlett Packard 6890 gas chromatograph (GC) interfaced with a Hewlett Packard 5973 mass selective detector. The GC was equipped with a split/splitless injector and a DB-5 capillary column (60 m × 0.25 mm i.d. coated with a 0.25 µm film thickness). The initial oven temperature (50 °C) was increased at a rate of 6 °C/min until reaching the final temperature (320 °C), initial and final hold times were 1 minute and 24 minutes, respectively. Ultra-high purity helium was used as a carrier gas at a constant flow (1.1 mL/min). The MS detector was operated at 70 eV (full scan) from 35–650 Da.

The detailed procedure used for maleimide purification is reported elsewhere^54^. In brief, polar fractions were purified by thin layer chromatography (TLC) using DCM:ethyl acetate (EtOAc) (4:1, v-v), along with a reference compound H,H maleimide (Sigma Aldrich) used as a retention standard. The band between retention factor (Rf) 0.6 and 0.9 (containing the maleimides) was recovered by elution with EtOAc over a small silica gel column.

Derivatisation with N-(tert-butyldimethylsilyl)-N-methyl trifluoroacetamide (MTBSTFA) in pyridine was performed to obtain tert-butyldimethylsilyl (TBDMS) derivatives of maleimides (e.g.^28,54^). TBDMS derivatives of maleimide in *n*-hexane were analysed by GC-MS using an Agilent HP 6890 GC system equipped with an Agilent DB-5MS column [60 m × 0.25 mm i.d. × 0.25 μm f.t.] coupled to an Agilent 5973 Mass Selective Detector operated at 70 eV. The temperature program for both instruments was 40 °C (1 min isothermal), 40 °C to 100 °C at 10 °C/min, 100 °C to 320 °C at 4 °C/min, isothermal at 320 °C for 30 min. Helium was used as carrier gas (1.2 mL/min). The maleimide was identified based on their mass spectrum, retention times and elution order by comparison with other published work (e.g.^29,53^).

CSIA was performed on a Thermo Finnigan Delta V mass spectrometer coupled to an Isolink GC. A pure cholesterol standard (underivatised and derivatised) was analysed in order to calculate the δ^13^C of the additional methyl-groups from BSTFA^54^. Samples were run as triplicates and the δ^13^C values of the parent compounds were corrected for the isotopic composition from the methyl-groups of the BSTFA^54^. A CO_2_ reference gas standard with a known δ^13^C value was introduced during CSIA to determine the δ^13^C values of the sterols. The δ^13^C are reported in per mil (‰) relative to the international Vienna Peedee Belemnite standard (VPDB); the values reported have a standard deviation below 0.4‰VPDB for at least 3 analyses.

### Availability of materials and data

The datasets generated during and/or analysed during the current study are available from the corresponding author on reasonable request.

## Electronic supplementary material

Supplementary information
