# Supplementary material for: Palaeobiology of red and white blood cell-like structures, collagen and cholesterol in an ichthyosaur bone
Source: Sci Rep. 2017 Oct 23;7:13776. doi: 10.1038/s41598-017-13873-4 (PMC5653768; doi:10.1038/s41598-017-13873-4)
Supplement: Supplementary file 1 — Supplementary information [file 41598_2017_13873_MOESM1_ESM.docx]

**Palaeobiology of red and white blood cell-like structures, collagen and cholesterol in an ichthyosaur bone**

Chloé Plet^1^, Kliti Grice^1*^, Anais Pagès^1,2^, Michael Verrall^2^, Marco J.L. Coolen^1^, Wolfgang Ruebsam^3^, William D.A Rickard^4^, Lorenz Schwark^1,3^

^1^ WA-Organic and Isotope Geochemistry, Department of Chemistry, The Institute for Geoscience Research, Curtin University, WA 6845, Australia

^2^ CSIRO CESRE, Mineral resources, Kensington, WA 6151, Australia

^3^ Department of Organic Geochemistry, Institute of Geoscience, Christian Albrechts University, Kiel 24118, Germany

^4^ Advanced Resource Characterisation Facility, John de Laeter Centre, Curtin University, WA 6845, Australia

*Correspondence to K.grice@curtin.edu.au

Table S1. Shows the total organic and inorganic carbon contents of the samples studied and the proportion of CaCO_3_, inferred from weight loss after acid treatment and from the total inorganic carbon (TIC) contents, assuming TIC is present as calcite only. The minor shift in values between the two methods is attributed to the higher uncertainty of the weight loss measurement; however, values strongly suggest TIC is contained in the calcite.

|  | TOC (%) | TIC (%) | CaCO_3_ (wt.%)  (by weight loss) | CaCO_3_ (wt.%)  (measured based on TIC) |
| --- | --- | --- | --- | --- |
| Bone | 0.23 | 7.1 | 55.6 | 59.1 |
| Concretion body | 1.13 | 10.5 | 90.4 | 87.5 |
| Concretion rim | 2.01 | 7.9 | 68.2 | 65.8 |


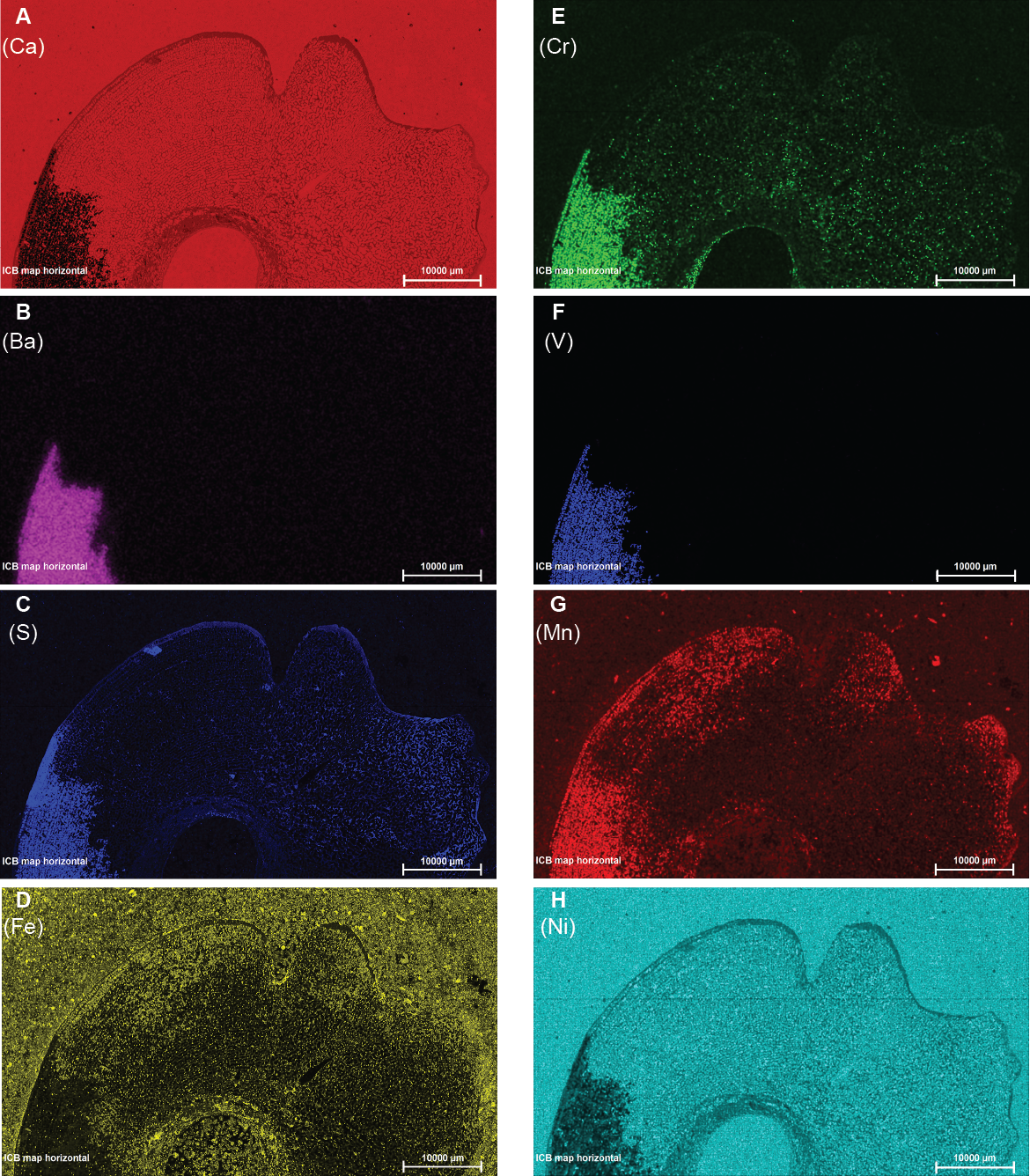


Figure S1. Microbeam XRF elemental mapping of a polished section of the concretion. A) Calcium (Ca) distribution map, showing high abundance of Ca in the concretion and bone porosity (bright red: Ca in calcite) and in the bone (dark red: Ca in fluorapatite). Locally, Ca is virtually absent from the pore space suggesting the presence of another mineral. B) Barium (Ba) distribution map. Ba signal is strongly located in the bone porosity where Ca is absent, which emphasises a different mineral filling that porosity. C) Sulfur (S) distribution. The distribution of S correlates with Ba suggesting that the mineral phase is barite (BaSO_4_). D) Iron (Fe) elemental distribution. Fe is widespread within the concretion but does not match the S distribution suggesting it is incorporated within the calcite. (E) to (H) distribution of various redox sensitive elements such as E) Chromium (Cr), F) vanadium (V), G) manganese (Mn), and H) nickel (Ni) clearly demonstrating the presence of different redox micro-environments within the bone porosity itself during the early stages of concretion precipitation.


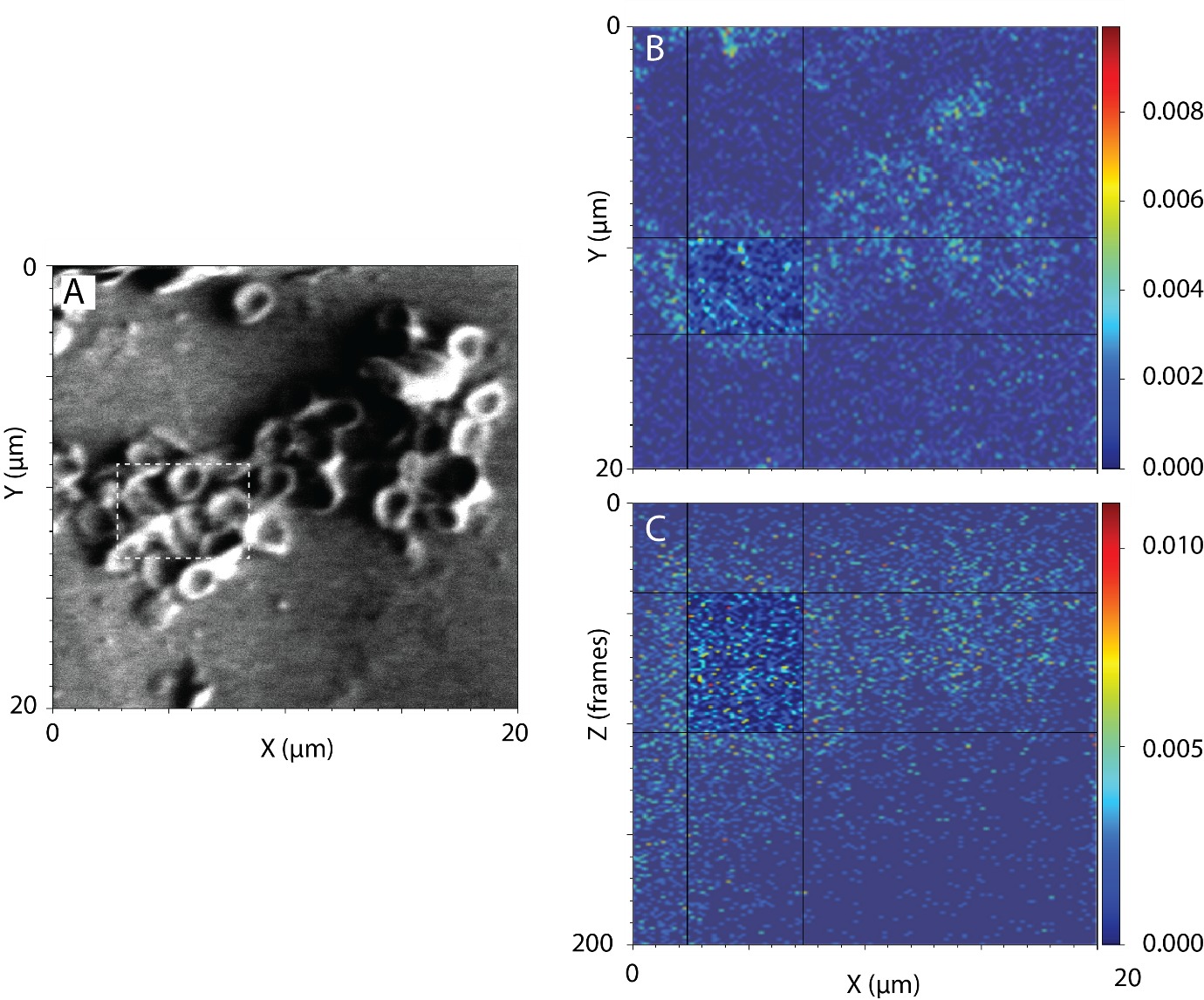


**Figure S2. ToF SIMS photomicrograph and analysis of the RBC-like structure.** A) Photomicrograph of the area where the mass spectra was acquired (Figure 5A). B) ^12^C SIMS map of a cluster of RBC-like structures. C) ^12^C SIMS map (side projection) of RBC-like structures. These ToF-SIMS images show higher C content between the surface and the substrate surface (consistent with C containing RBC-like structures).


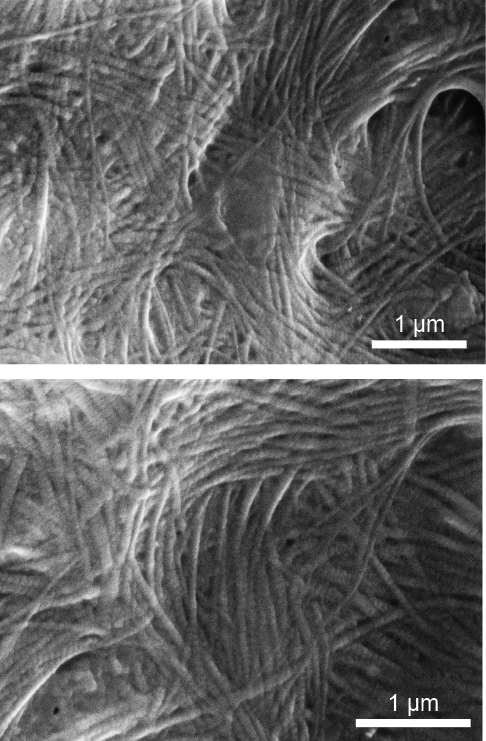


Figure S3. SEM images of collagen fibres in a modern crocodile bone. Abundant flexible fibres that show darker banding, characteristic of collagen, were observed. These images display strong morphological similarities with the collagen-like fibres identified in the fossil ichthyosaur bone.
